# Influence of bevacizumab, sunitinib and sorafenib as single agents or in combination on the inhibitory effects of VEGF on human dendritic cell differentiation from monocytes

**DOI:** 10.1038/sj.bjc.6604965

**Published:** 2009-03-10

**Authors:** C Alfaro, N Suarez, A Gonzalez, S Solano, L Erro, J Dubrot, A Palazon, S Hervas-Stubbs, A Gurpide, J M Lopez-Picazo, E Grande-Pulido, I Melero, J L Perez-Gracia

**Affiliations:** 1 Gene Therapy and Hepatology Division, CIMA, Universidad de Navarra, Pamplona 31008, Spain; 2 Biochemistry Department, Clínica Universitaria de Navarra, Universidad de Navarra, Pamplona 31008, Spain; 3 Medical Oncology Department, Clínica Universitaria de Navarra, Universidad de Navarra, Pamplona 31008, Spain;; 4 Clinical Research Department, Pfizer Inc., Madrid 28006, Spain; 5 Internal Medicine Department, Clínica Universitaria de Navarra, Universidad de Navarra, Pamplona 31008, Spain

**Keywords:** dendritic cells, renal cell carcinoma, VEGF, bevacizumab, sunitinib, sorafenib

## Abstract

Vascular endothelial growth factor (VEGF) inhibits differentiation and maturation of dendritic cells (DC), suggesting a potential immunosuppressive role for this proangiogenic factor. Bevacizumab, sorafenib and sunitinib target VEGF-mediated angiogenesis and are active against several types of cancer, but their effects on the immune system are poorly understood. In this study, VEGF and supernatants of renal carcinoma cell lines cultured under hypoxia were found to alter the differentiation of human monocytes to DC. Resulting DC showed impaired activity, as assessed by the alloreactive mixed T-lymphocyte reaction. Bevacizumab and sorafenib, but not sunitinib, reversed the inhibitory effects of VEGF, but not of those mediated by tumour supernatants. Dendritic cells matured under the influence of VEGF expressed less human leukocyte antigen-DR (HLA-DR) and CD86, and this effect was restored by bevacizumab and sorafenib. Finally, tumour-cell supernatants decreased interleukin-12 (IL-12) production by mature DC, and such inhibition was not restored by any of the tested drugs, delivered either as single agents or in combination. The deleterious effects of tumour-cell supernatants were mainly mediated by thermostable molecules distinct from VEGF. These results indicate that inhibition of the differentiation of monocytes to DC is a multifactorial effect, and that they support the development of combinations of angiogenesis inhibitors with immunological modulators.

Angiogenesis is a critical step in the development of solid tumours and their metastasis, and its blockade has become a major target of cancer therapy (Kerbel and Folkman, 2002). Renal cell carcinoma (RCC) was the first human tumour in which inhibition of angiogenesis, mediated by bevacizumab, a monoclonal antibody (mAb)-neutralising vascular endothelial growth factor (VEGF), showed clinical benefit (Yang *et al*, 2003a). Subsequently, RCC has remained a platform for the development of antiangiogenic compounds. Sunitinib, a tyrosine kinase inhibitor of VEFG receptor (VEGFR), c-kit and platelet-derived growth factor receptor, was superior to interferon-*α* (IFN-*α*) in a randomised phase III trial as first line treatment of RCC (Motzer *et al*, 2007), and sorafenib, another tyrosine kinase inhibitor that targets *Ras*-activated factor (RAF) and VEFGR, also showed clinical benefit in a phase III trial in which it was compared with placebo as second-line treatment of this disease (Escudier *et al*, 2007a). Bevacizumab, in combination with IFN, was also superior to single agent IFN in another phase III trial (Escudier *et al*, 2007b).

Even though the main mechanism of the action of drugs that inhibit VEGF-related pathways is antiangiogenesis, an effect of VEGF inhibitors on the immune system has been reported. In an earlier study, Gabrilovich *et al* (1996) obtained dendritic cells (DC) from umbilical cords and described an inhibition of their ability to induce T-lymphocyte proliferation, assessed by the mixed lymphocyte reaction (MLR), when they were matured in supernatant cell cultures containing VEGF. This effect was partially reverted by anti-VEGF antibodies, showing that VEGF was probably the cause of inhibition of DC-induced proliferation. The same authors showed that VEGF inhibited the development of DC and increased B lymphocytes and immature myeloid cells in animal models (Gabrilovich *et al*, 1998). The proposed mechanism of action was the interference with the activation of nuclear factor-*κ*B transcription factors (Ohm *et al*, 1999). Using the same model, this group also showed that the addition of anti-VEGF antibodies increased the number and activity of DC in mice implanted with subcutaneous tumours (D459 and MethA sarcoma), although without any therapeutic effect on tumour growth (Gabrilovich *et al*, 1999). Nevertheless, a combined treatment with anti-VEGF antibodies and DC pulsed, with p53 peptides that contained specific mutations of the implanted tumours, produced a sustained antitumour effect, indicating potential synergism between antiangiogenic drugs and DC-based immunotherapy treatments for cancer (Gabrilovich *et al*, 1999). Other groups have suggested that angiogenic factors at the tumour microenvironment differentiate DC that display features of vascular cells and are immunosuppressive (Conejo-Garcia *et al*, 2004). In addition, VEGF has been described to mediate immunosuppression (Ohm *et al*, 2003), which depends on the inhibition of differentiation and migration of thymic lymphocyte progenitors from the bone marrow and subsequent reduction of the numbers of CD4^+^ and CD8^+^ thymocytes, which can be reverted when exposure to VEGF ceases. Cancer therapeutic vaccines formulated with DC artificially presenting tumour antigens are being extensively tested in clinical trials in several tumour types (Vulink *et al*, 2008), including RCC (Dannull *et al*, 2005). The possibility to overcoming potential interactions of VEGF on the immune system through clinically available angiogenesis inhibitors led us to evaluate the influence of such drugs on DC differentiation from monocytes. A recent report has documented the inhibitory effects of sorafenib, but not of sunitinib, on DC maturation (Hipp *et al*, 2008). Maturation is the gene expression programme that renders DC capable of mediating T-cell expansion and activation (Steinman, 2008). Maturation is triggered by microbial biomolecules (such as, lipopolysaccharide (LPS) or bacterial DNA), proinflammatory cytokines and ongoing T-cell responses. In spite of the reports on the effects of VEGF on DC, little is known about the effects of these compounds on the differentiation of DC from myeloid precursors. In this study, the effects of soluble VEGF and of VEGF-containing supernatants from RCC cells were assessed on monocytes to DC differentiation cultures. The influence of bevacizumab, sorafenib and sunitinib on such cultures was defined.

## Materials and methods

### DC generation and maturation

Dendritic cells were generated from filter buffy coats (FBC)-derived monocytes donated by healthy human donors (Meyer *et al*, 2005). The protocol for obtention of FBC was approved by the Ethics Committee of our institution and was carried out on donors who gave informed consent. Isolated mononuclear cells were subjected to positive selection using anti-CD14-conjugated paramagnetic beads and purified using the AutoMACS system according to the manufacturer's instructions (Miltenyi Biotec, Bergisch Gladbach, Germany). Purified monocytes were cultured for 7 days in AIM-V serum-free media (Gibco-BRL, Gaithersburg, MD, USA), supplemented with granulocyte-macrophage colony-stimulating factor (GM-CSF) (1000 U ml^−1^; Leukine, Berlex, Richmond, CA, USA) and interleukin-4 (IL-4, 500 U ml^−1^; R&D Systems, Minneapolis, MN, USA). Cytokines were added every 2 days. Dendritic cells were matured with clinical-grade tumour necrosis factor-*α* (TNF-*α*; 50 ng ml^−1^; Boehringer Ingelheim, Ingelheim, Germany), IFN-*α* (1,000 IU ml^−1^; Schering-Plough, Kenilworth, NJ, USA) and poly I:C (20 *μ*g ml^−1^; Ampligen, Bioclones, Tokai, South Africa) for 48 h.

Maturation was confirmed by assessing increases in the immunofluorescence of CD80, CD83, CD86 and human leukocyte antigen-DR (HLA-DR), as well as variations in CD1a. Flow cytometric analysis for immature and mature DC was performed at days 7 and 9, using a FACSCalibur Flow Cytometer (Becton Dickinson, San Diego, CA, USA). Purified anti-VEGFR-1 and VEGFR-2 mAbs were purchased from Santa Cruz Biotechnology (Santa Cruz, CA, USA) and purified anti-VEGFR-3 mAbs from R&D Systems. Indirect immunofluorescence was performed with anti-mouse IgG1-FITC (Dako, Glostrup, Denmark). Cell viability was confirmed by the trypan blue exclusion test. Recombinant human VEGF 165 (25 ng ml^−1^, R&D Systems), bevacizumab (rhumAb-VEGF, Avastín, 1 *μ*g ml^−1^, Roche, Basel, Switzerland), sorafenib (Nexavar, 10 ng ml^−1^, Bayer, Berlin, Germany) and sunitinib (Sutent, 10 ng ml^−1^, Pfizer, New York, NY, USA) were used during DC differentiation. For experiments with supernatant of RCC line cultures, we added 1 : 5 (v/v) ratio of the RCC-10 supernatant to the culture medium of DC.

### Cell culture and reagents

Parental von Hippel–Lindau (VHL)-negative RCC-10 cells, originally cultured from a clear cell renal adenocarcinoma case (Krieg *et al*, 2000; Cuevas *et al*, 2003), and a clone derived by stable transfection of the VHL gene (VHL+53), were kindly provided by Dr Luis del Peso (CSIC-UAM, Madrid, Spain). The cells were maintained in an RPMI 1640 medium with GLUTAMAX-I (Invitrogen, Carlsbad, CA, USA). The culture medium was supplemented with 100 units ml^−1^ penicillin, 100 *μ*g ml^−1^ streptomycin and 10% foetal bovine serum. To perform hypoxic experiments, hypoxia was induced by exposing cell cultures to 1% oxygen for 24–48 h, using a hypoxic workstation (Cuevas *et al*, 2003).

### MLR

Dendritic cells were cultured in 96-well plates in an AIM-V medium. A total of 2 × 10^5^ lymphocytes from a distinct donor were added on day 9 at different Tcell/DC ratios (80 : 1, 40 : 1 and 20 : 1). After 3 days, the [methyl-^3^H]thymidine uptake was determined by the addition of 1 *μ*Ci of [methyl-^3^H]thymidine (25 Ci mmol^−1^; Amersham, Uppsala, Sweden) for 16–20 h. At the end of this labelling time, the [methyl-^3^H]thymidine uptake was determined by transferring cells to 96-well filter microplates (Unifilter-96 GF/C, PerkinElmer, Boston, MA, USA) and adding 25 *μ*l of liquid scintillation (Microscint O, PerkinElmer) to measure radioactivity. Technical controls were from phytohemagglutinin (PHA)-stimulated human lymphocyte populations from a single individual.

### Cytokine measurement

Renal cell carcinoma culture supernatants (10^6^ cells/ml) were assayed for VEGF production at 24 and 48 h with an enzyme-linked immunoabsorbent assay (ELISA) kit (R&D Systems). Interleukin-6, IL-8, IL-10, TNF-*α* and IL-12 were simultaneously analysed by microparticle-based flow cytometry (Cytometric Bead Array) in supernatant samples of DC cultures at baseline and on day 2, according to the manufacturer's instructions (BD Bioscience, San Jose, CA, USA).

### Indoleamine 2,3-Dioxygenase (IDO) activity measurement

Indoleamine 2,3-Dioxygenase (IDO) activity in DC culture supernatants was measured by high-performance liquid chromatography (HPLC). The samples were deproteinised by mixing 100 *μ*l supernatant and 100 *μ*l of 30% (w/v) trichloroacetic acid. After centrifugation (4 min at 10 000 **g**), 25 *μ*l of the supernatant was added to a vial containing Na_2_HPO_4_ buffer of 125 *μ*l (HPLC: Hewlett-Packard Series 1100; Agilent Technologies Inc., Santa Clara, CA, USA) using a 5 *μ*Mcolumn C18 Symmetry Shield (Waters Corporation, Milford, MA, USA). The liquid chromatography parameters were injection volume: 100 *μ*l; flow: 0.8 ml min^−1^; stop time: 15 min; maximum pressure: 350 bar. The mobile phase was 15 mM sodium acetate (pH 4) with 27 ml l^−1^ acetonitrile. The IDO activity is proportional to the [kynurenine]/[tryptophan] ratio. Kynurenine concentration was measured at 360 nm and tryptophan at 285 nm, with a spectrophotometer coupled to HPLC. A standardised curve to measure kynurenine and tryptophan concentrations was plotted and assessed.

### Arginase activity assay

Cell lysates were tested for arginase activity by measuring the production of urea derived from L-arg. A volume of 100 *μ*l of cell lysate was treated with Cell Lysis Buffer (Cell Signaling Technology, Danvers, MA, USA). The resulting lysate was added to 100 *μ*l of an activation solution (Tris-HCl 25 mM, pH 7.4, MnCl_2_ 5 mM). The mixture was heated for 10 min at 56°C, mixed with 100 *μ*l of 0.5 M arginine and incubated for 30 min at 37°C. The reaction was interrupted with 200 *μ*l of an acid solution (H_2_SO_4_/H_3_PO_4_/H_2_O; 1 : 3: 7 (v/v/v)) and the samples were boiled for 45 min with 20 *μ*l of 9% (v/v) *α*-isopropiophenone in absolute ethanol. The enzymatic activity was assessed by colorimetric reaction in a spectrophotometer at 540 nm using a standard curve.

### Fractions of RCC by dialysis and filtration

Renal cell carcinoma supernatant was fractionated to determine the molecular weight of the inhibitory molecules using centrifugal filters (Microcon, Millipore, Billerica, MA, USA) of 10, 30, 50 and 100 kDa. The collected fractions were tested on monocytes differentiating to DC by incubation with IL-4 and GM-CSF for 7 days. The only fraction that did not produce the inhibition of full differentiation of DC from monocytes was below 10 kDa. To know the more exact the molecular weight, the RCC supernatant was included in a 3.5 kDa dialysis cassette (Slide-A-Lycer Dialysis Cassette; 3500 MWCO; Pierce, Rockford, IL, USA) for 2 h at room temperature and overnight at 4°C under continuous stirring. Thereafter, the supernatant with moieties of molecular weight over 3.5 kDa was fractionated using the same process. To assess thermostability of the putative inhibitory factor, RCC supernatants were boiled at 100°C for 5 min.

## Results

### RCC culture supernatants contain abundant VEGF that is increased by hypoxia

**Figure 1:**
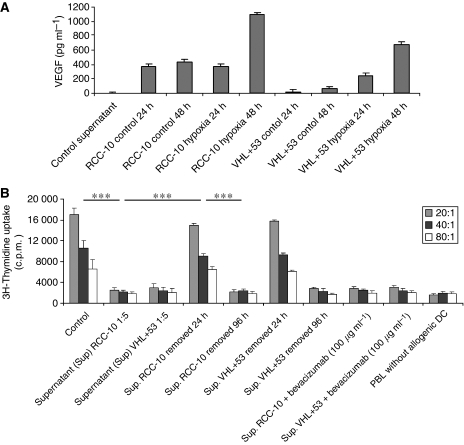
Tissue culture supernatants from RCC cells contain VEGF and suppress differentiation of MLR-stimulating DC from monocytes. (**A**) Vascular endothelial growth factor concentrations measured by ELISA in the 24 and 48 h conditioned media from ∼80% confluent cultures of the indicated cell lines. Renal cell carcinoma-10 is a renal cell carcinoma with VHL deficiency. VHL+53 is a stable transfectant recovering the VHL expression. Cultures were carried out in normoxia (21% O_2_) or hypoxia chambers (1% O_2_) as indicated. HT-29 human colon carcinoma cell supernatants were used as control supernatant. (**B**) Lymphocyte proliferation at the indicated DC to allogenic PBL ratios, as induced by DC differentiated for 7 days from monocytes in the presence of GM-CSF and IL-4 with or without 1 : 5 (v/v) of the indicated conditioned media. After differentiation, DC were matured for 48 h with TNF-*α*, IFN-*α* and poly I:C. Conditioned media were removed when indicated by three washes. Anti-VEGF mAb (bevacizumab) was added at the indicated concentrations for the duration of the differentiation culture. Mature DC differentiated without additives were used as positive control. Data represent mean±s.d. from three experiments. ^***^P<0.001, statistical analyses were performed by the Kruskal–Wallis statistical test.

Vascular endothelial growth factor levels were measured in the culture supernatant from RCC-10 and VHL+53 tumour cell lines under normal and hypoxic (1% O_2_) conditions (Figure 1A). Renal cell carcinoma-10 lacks VHL expression, whereas VHL+53 is a transfected revertant for this gene that controls the hypoxia response. Exposure to hypoxia for 48 h stimulated VEGF secretion in both cell lines. Vascular endothelial growth factor production under hypoxia was higher by RCC-10 than by VHL+53, despite the absence of the VHL expression. The supernatant of RCC-10 under 48 h hypoxia was chosen for subsequent experimentation.

### Human DC differentiated in the presence of VEGF or RCC supernatants show impaired ability to induce T-cell proliferation, as assessed by MLR

**Figure 2:**
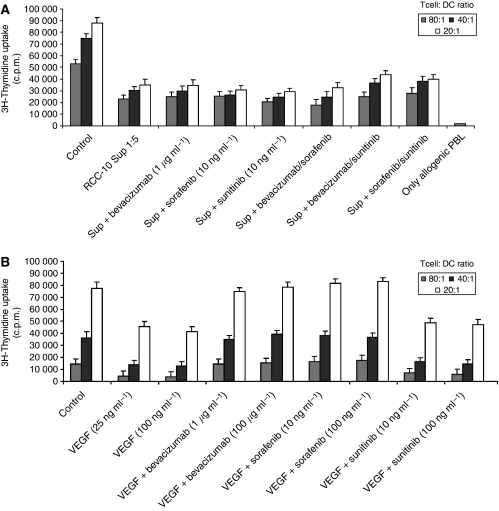
Vascular endothelial growth factor and RCC supernatants during monocyte differentiation to DC inhibit MLR-stimulating activity of resulting DC. Vascular endothelial growth factor-mediated inhibition is reversible by bevacizumab and sorafenib. (**A**) CD14+ monocyte cultures as in Figure 1 were set up in the presence of VEGF and the indicated VEGF inhibitors. In all the experiments, TNF-*α*, IFN-*α* and poly I:C were added to induce DC maturation for 48 h, including the control culture. (**B**) Anti-VEGF agents in different combinations are tested at the indicated concentrations to reverse the inhibition in the MLR from mature DC that had been differentiated in the presence of RCC supernatants. Mature DC without VEGF or RCC supernatants were used as positive controls. Microcultures of allogenic PBL without DC are plotted as negative controls. Results in panels a and b represent the mean±s.d. from four different experiments.

Dendritic cells were differentiated from CD14^+^ monocytes for 7 days by GM-CSF and IL-4 in the presence of either supernatants from RCC cultured under hypoxic conditions (Figure 1B) or from VEGF (Figure 2A), leading to a strong inhibition of their ability to induce allogenic T-cell proliferation, as determined by the primary MLR. This inhibition did not take place if tumour-cell supernatants were removed at 24 h, but remained if supernatants were washed after 96 h of culture (Figure 1B). The supernatants from other cell lines in Figure 1 showed similar effects (data not shown).

### Bevacizumab and sorafenib reverse the effects induced by VEGF on DC activity. Neither of the drugs, as single agents or in combination, reversed the inhibitory effects of RCC culture supernatants

The addition of bevacizumab or sorafenib restored the MLR of DC differentiated in the presence of VEGF to baseline levels, whereas sunitinib did not (Figure 2A). As the activity of indoleamine 2,3-dioxygenase (Munn *et al*, 2002) or arginase (Ochoa *et al*, 2007) can decrease the ability of DC to stimulate T cells, we tested the activity of both enzymes in DC differentiated under the effects of VEGF, without finding evidence of increased activity even in the presence of pharmacological antagonists (Supplementary Figure 1).

The decrease in MLR stimulating activity caused by RCC culture supernatants could not be restored by bevacizumab, sorafenib or sunitinib, delivered either as single agent or in combination (Figures 1B and 2B). Therefore, it can be concluded that VEGF is not the only or main mediator of the deleterious effect played by RCC supernatants on DC differentiation from monocytes.

### VEGF and RCC supernatants induce changes in the surface phenotype of DC

**Figure 3:**
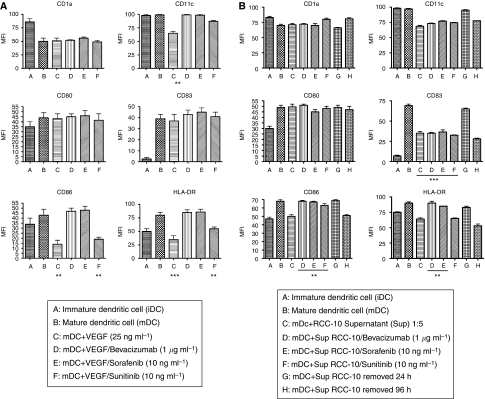
Vascular endothelial growth factor and RCC supernatants added to DC differentiation cultures inhibit maturation-induced surface markers on DC: effects of bevacizumab, sorafenib and sunitinib. Dendritic cells obtained from monocytes in 7-day cultures with GM-CSF and IL-4 were analysed by surface immunostaining and flow cytometry for the indicated leukocyte differentiation antigens before and after maturation in the presence of TNF-*α*, IFN-*α* and poly I:C. As indicated, recombinant VEGF (**A**) or RCC supernatants (**B**) were added during differentiation. When indicated, bevacizumab, sorafenib or sunitinib was also added during the differentiation cultures. Values are expressed as mean±s.e.m. of four different experiments. ^**^P<0.01,^***^P<0.001, statistical analyses were performed by the Kruskal–Wallis statistical test. In all cultures set up with CD14^+^ cells, the expression of CD14 was lost, so at the end of the cultures <1% cells were CD14^+^.

We determined the phenotype of DC differentiated with and without recombinant VEGF or RCC supernatants. Dendritic cells were maturated with a cocktail containing TNF-*α*, IFN-*α* and poly I:C (all manufactured under good manufacturing practice conditions). We assessed the surface expression of CD1a, CD11c, CD80, CD83, CD86, HLA-DR and CD14 using flow cytometry analyses (Figures 3A and B). The most relevant effects induced by VEGF on mature DC were marked decreases in the intensity of CD11c, CD86 and HLA-DR. These effects were completely reversed by the addition of bevacizumab and sorafenib. Sunitinib also restored the normal expression of CD11c, but not of CD86 and HLA-DR. Supernatants from RCC decreased the expression intensity of CD11c, CD83, CD86 and HLA-DR to some extent . In these cases, bevacizumab and sorafenib restored CD86 and HLA-DR but not CD83. Renal cell carcinoma supernatants, but not VEGF, led to DC cultures in which cells were more adherent and presented spread morphology, which was more reminiscent of macrophages under phase contrast-microscopy (Supplementary Figure 2).

It is of note that DC differentiated with VEGF did not repress the MLR stimulating activity when added to third-party DC/T cell cultures, thus revealing the fact that VEGF yields less-active DC rather than suppressive DC (Supplementary Figure 3).

### Addition of VEGF or RCC supernatants during DC maturation did not alter the MLR-promoting activity of DC

**Figure 4:**
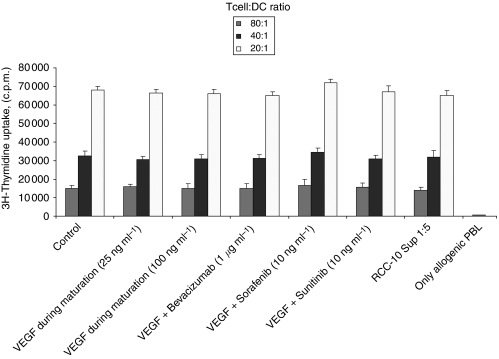
Vascular endothelial growth factor and RCC supernatants, when added during maturation, cannot inhibit the MLR-stimulating activity of already differentiated DC. Vascular endothelial growth factor and RCC supernatants were added to differentiated DC during the 48 h maturation culture with TNF-*α*, IFN-*α* and poly I:C. When indicated, VEGF-inhibiting drugs were also added. The mitogenic activity on allogenic T cells of DC from the different conditions was monitored. Mature DC without further additives were used as positive control.

In contrast to monocyte to DC differentiation cultures, neither VEGF nor RCC supernatants altered the MLR-stimulating activity of DC if they were added to the culture during the 48 h of maturation as induced by the combined effects of TNF-*α*, IFN-*α* and poly I:C (Figure 4). This indicates that once differentiated, DC are less sensitive to the effects of VEGF or RCC supernatants with regard to activation/maturation, at least when triggered by these powerful maturation-inducing agents.

### RCC culture supernatants, but not VEGF, decrease the production of IL-12 by mature DC

**Figure 5:**
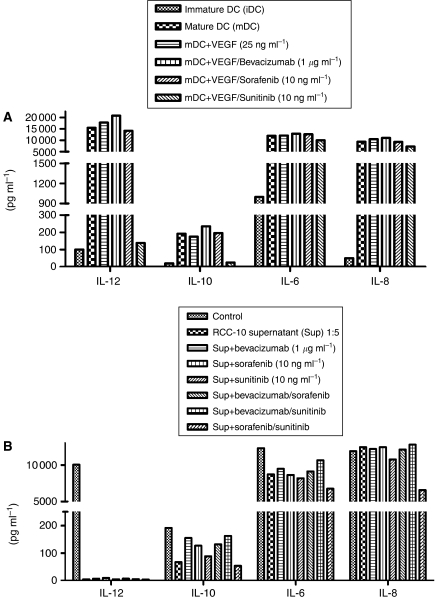
Dendritic cell differentiation in the presence of RCC supernatants or sunitinib shows less IL-12 production. Cytokines were measured by CBA assays in the supernatant of the indicated DC cultures treated for the length of their differentiation from monocytes as indicated. All the experiments were performed with mature DC except when indicated as immature DC. (**A**) The effect of VEGF and drug inhibitors is shown, whereas the effect of RCC supernatants, along with bevacizumab, sorafenib, sunitinib or its combinations, at the indicated concentrations is shown (**B**).

We determined concentrations of IL-12, IL-10, IL-6 and IL-8 in cultures of immature and mature DC, using microparticle-based flow cytometry assays. It is interesting that the presence or absence of VEGF during differentiation did not change the production of IL-6, IL-8, IL-10 and IL-12. It is surprising that the addition of sunitinib during DC differentiation dramatically reduced IL-12 and, to a lesser extent, IL-10 concentrations (Figure 5A and Supplementary Figure 4, showing dose dependency). Such effects did not occur when DC differentiation cultures were exposed to either bevacizumab or sorafenib, suggesting a mechanism of action that is dependent not on VEGF-R but rather on an alternative pathway (Figure 5A).

We also measured the same cytokines on DC from cultures either conditioned or not with (1 : 5 (v/v)) hypoxic renal carcinoma cell-culture supernatant. The supernatant from the cell lines inhibited IL-12 production almost completely, and such inhibition was maintained in spite of the addition of antiangiogenic drugs. The supernatant did not alter the levels of the other assessed cytokines (Figure 5B), further indicating the functional viability of the cultures and suggesting immunomodulatory effects.

### The effects of RCC culture supernatants on DC activity are mainly mediated by small thermostable molecule/s and cannot be reversed by VEGF pharmacological antagonism

As we could not revert the inhibition of MLR activity caused by RCC culture supernatants with either of the antiangiogenic compounds, we fractionated the RCC-10 supernatant to determine the molecules that could cause this effect.

**Figure 6:**
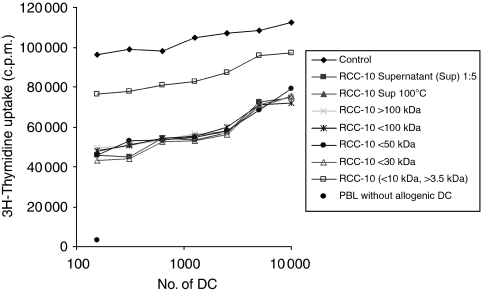
The inhibitory activity of RCC-10 supernatants on the differentiation of MLR-stimulating mature DC is mainly mediated by thermostable molecules in the range of 3.5–10 kDa. RCC-10 was used as crude or fractionated by filtration and/or dialysis in molecules of the indicated molecular weight ranges. The inhibitory activity was present after treating RCC-10 supernatants for 5 min at 100°C. Mature DC without additives under identical conditions were used as positive controls for MLR activity, and microcultures of allogenic lymphocytes without DC are plotted as negative controls. The experiment shown is representative of the three similarly performed.

Filtration fractions showed that small molecule/s of <10 kDa suppressed the MLR activity of DC (Figure 6). Further fractionation by dialysis pre-excluding molecules <3.5 kDa showed that the activity was partially recovered when molecules >3.5 kDa were excluded. Accordingly, most of the inhibitory activity can be traced to the 3.5–10 kDa range. Moreover, such inhibitory activity persisted after heating the supernatant for 5 min at 100°C (Figure 6).

These results conclude that small soluble molecules distinct from VEGF (described as a moiety in the 35–45 kDa range) are the main mediators of decreased T-cell-stimulating activity in DC differentiated from monocytes in the presence of RCC culture supernatants.

## Discussion

The treatment of metastatic kidney cancer has rapidly evolved during the last few years, and a large number of targeted molecules have either already shown efficacy in phase III trials or are in advanced stage of clinical development, replacing former immunotherapeutic approaches (Schrader and Hofmann, 2008). Nevertheless, immunotherapy may retain a role in the treatment of this disease. It must be remembered that the only treatment that can induce cure in some patients with metastatic renal cell cancer is high-dose intravenous IL-2 (Yang *et al*, 2003b; McDermott *et al*, 2005). Moreover, immunotherapy with vaccines is the only treatment that has shown clinical efficacy as an adjuvant treatment of resected RCC (Jocham *et al*, 2004). Finally, a large number of treatments based on the modulation of immunotherapy are being developed (Melero *et al*, 2007) and some of them are being tested in patients with metastatic RCC, suggesting that, in the near future, combinations of targeted agents with new immunotherapeutic approaches might become a reality (Coppin *et al*, 2008). In addition, as mentioned earlier, VEGF interferes with the development and function of DC (Gabrilovich *et al*, 1996, 1998), and such an effect can be blocked by anti-VEGF antibodies. Therefore, the study of potential interactions between targeted agents currently used in the treatment of RCC and the immune system is of interest.

Our data confirm that VEGF inhibits the functional differentiation of DC, and impairs their ability to induce allogenic T-cell proliferation as shown by the decrease in the allogenic MLR assay. Such an inhibition was not dependent on indoleamine 2,3-dioxygenase or on arginase activity factors, which have been described as immunosuppressive in DC and other myeloid cells (Munn *et al*, 2002; Ochoa *et al*, 2007). The addition of either sorafenib or bevacizumab reverted the effects of VEGF on DC. Sunitinib did not restore the ability of DC to induce a normal MLR, despite the fact that the concentrations used were in the range as those observed in cancer patients (31.8–65.9 ng ml^−1^) (Faivre *et al*, 2006). The immune phenomena of these kinds in RCC patients might be crucial.

To mimic *in vivo* conditions, we assessed the effects of differentiating DC in the presence of VEGF-containing supernatants from RCC cell lines cultured under hypoxic conditions. The supernatants induced a strong inhibition of DC differentiated in such a manner that it was not reverted by the addition of any of the anti-VEGF tested drugs. Moreover, combinations of the drugs did not have any restoring effects on the MLR decrease induced by the supernatants. Fractionation of those supernatants showed that small molecule/s >3.5 kDa and <10 kDa suppressed the MLR activity of DC, and that such molecules were thermostable. For this reason, metabolic products, such as tryptophan metabolites, can also be excluded as being responsible for the suppression. These results indicate that tumour cells exert their inhibitory function on differentiating DC through soluble factors distinct from VEGF, although VEGF could also be involved to a lesser extent.

Changes in DC differentiated in the presence of recombinant VEGF or RCC supernatants included marked decreases in CD11c, CD86 and HLA-DR expressions, which were completely reverted by bevacizumab and sorafenib, whereas sunitinib only restored normal expression of CD11c. The events in the transition from monocyte to DC are important not only because they represent a popular method of generating DC for immunotherapy (Vulink *et al*, 2008) but also because they might resemble the physiological differentiation of DC from myeloid precursors. The effect of VEGF on monocytes is not surprising because these leukocytes express abundant levels of the three identified receptors for this cytokine (Supplementary Figure 5). Moreover, during differentiation culture to DC, VEGFRs are downregulated.

It is interesting that, neither VEGF nor RCC supernatants were able to inhibit MLR if they had been added only during DC maturation/activation, rather than during the 7-day differentiation culture from monocytes. This indicates that mature DC might be more resistant to the effects of RCC supernatants or VEGF. Alternatively, the good manufacturing practice-compliant maturation cocktail that we use may be of such a strength that is not amenable to inhibition by these agents.

Exposure to VEGF during differentiation did not alter the secretion of several cytokines, including that of IL-12, IL-10, IL-6 and IL-8. The addition of sunitinib during DC differentiation induced a marked decrease in IL-12 concentrations and a less relevant reduction in IL-10 secretion. Bevacizumab and sorafenib did not reproduce these effects. These results suggest that the IL-12 inhibition induced by sunitinib is not mediated by the VEGF pathway, and may have relevant implications when exploring combinations of sunitinib with other immunotherapeutic strategies. Indeed, sunitinib and sorafenib are far from being specific inhibitors of a single tyrosine kinase and have been shown to inhibit various kinases with different ICs_50_. These included signalling pathways known to be critical in leukocyte biology (Karaman *et al*, 2008). Therefore, this type of VEGF-R-independent effects is not unexpected to see. Renal cell carcinoma culture supernatants also inhibited IL-12 production, and neither of the drugs tested reversed this effect, further supporting the fact that IL-12 inhibition is dependent not on the VEGF pathway but on other unidentified factors. Interleukin-12 is critical for therapeutic immunity against cancer (Mazzolini *et al*, 2003). Artificial enhancement of IL-12 production by DC strengthens elicited immunity and therapeutic potential (Melero *et al*, 1999; Mazzolini *et al*, 2005). Therefore, selective inhibition of IL-12 is likely to be suppressive for cellular immune responses.

Other authors have also evaluated the effects of sorafenib and sunitinib on DC (Hipp *et al*, 2008). They reported that sorafenib, but not sunitinib, inhibited DC function, assessed through the expression of cytokines and CD1a. The inhibitory effects were mediated by the inhibition of phosphatidylinositide 3-kinase production and mitogen-activated protein kinases, as well as by nuclear factor-*κ*B signalling. They treated C57BL/6 mice with both drugs, and found that sorafenib significantly reduced the induction of antigen-specific T cells, whereas sunitinib reduced the number of regulatory T cells in peripheral blood. On the basis of these findings, they concluded that sorafenib does not seem to be a good candidate for combination with immunotherapeutic approaches, whereas sunitinib does. The main difference between both studies is that whereas Hipp *et al* (2008) investigated the effects on already differentiated DC, we focused on the transition from myeloid immature precursors to DC.

Our experimental observations join the literature on potential mechanisms accounting for immune dysfunction in RCC. Direct proapoptotic effects of RCC cells on human T cells have been reported as being mediated by the FasL expression and gangliosides released into the surrounding media (Das *et al*, 2008). Gangliosides are unlikely mediators of the effects on monocytes because of lower molecular weights than that of the expected factors according to Figure 6. In RCC patients, sunitinib treatment improves type I responses by T lymphocytes with decreases in regulatory T cells (Finke *et al*, 2008). Whether this is mediated directly on T cells within the cancer-modified environment or by indirect effects involving myeloid leukocytes, such as DC, remains to be seen. The effects of sunitinib downmodulating the ability for IL-12 production in our cultures are to be reconciled with the improvements of type I responses seen in the patients and possibly related to the complexity of the *in vivo* system (Finke *et al*, 2008).

In conclusion, our study shows that bevacizumab and sorafenib, but not sunitinib, reverse the inhibitory effects of recombinant VEGF on DC differentiation, but not of those mediated by RCC culture supernatants, which are mainly mediated by other unidentified substance(s). The changes induced by VEGF on mature DC included a reduced expression of HLA-DR and CD86, which was also reverted by bevacizumab and sorafenib. Finally, RCC culture supernatants and, surprisingly, sunitinib decreased IL-12 production by mature DC. Such inhibition was not restored by any of the tested drugs, delivered either as single agents or in combination. Moreover, our data indicate that soluble factors from RCC exert effects on monocyte to DC differentiation, which renders functionally impaired DC for T-cell stimulation. Fortunately, such altered DC by tumour-soluble factors do not interfere with the function of healthy DC differentiated apart from the influence of tumour cells. Biochemical identification of the inhibitory factors is the next step to progress in our understanding of the new described phenomena.
