# Supplementary figures and images for: Influence of bevacizumab, sunitinib and sorafenib as single agents or in combination on the inhibitory effects of VEGF on human dendritic cell differentiation from monocytes
Source: Br J Cancer. 2009 Mar 10;100(7):1111–9. doi: 10.1038/sj.bjc.6604965 (PMC2670006; doi:10.1038/sj.bjc.6604965)

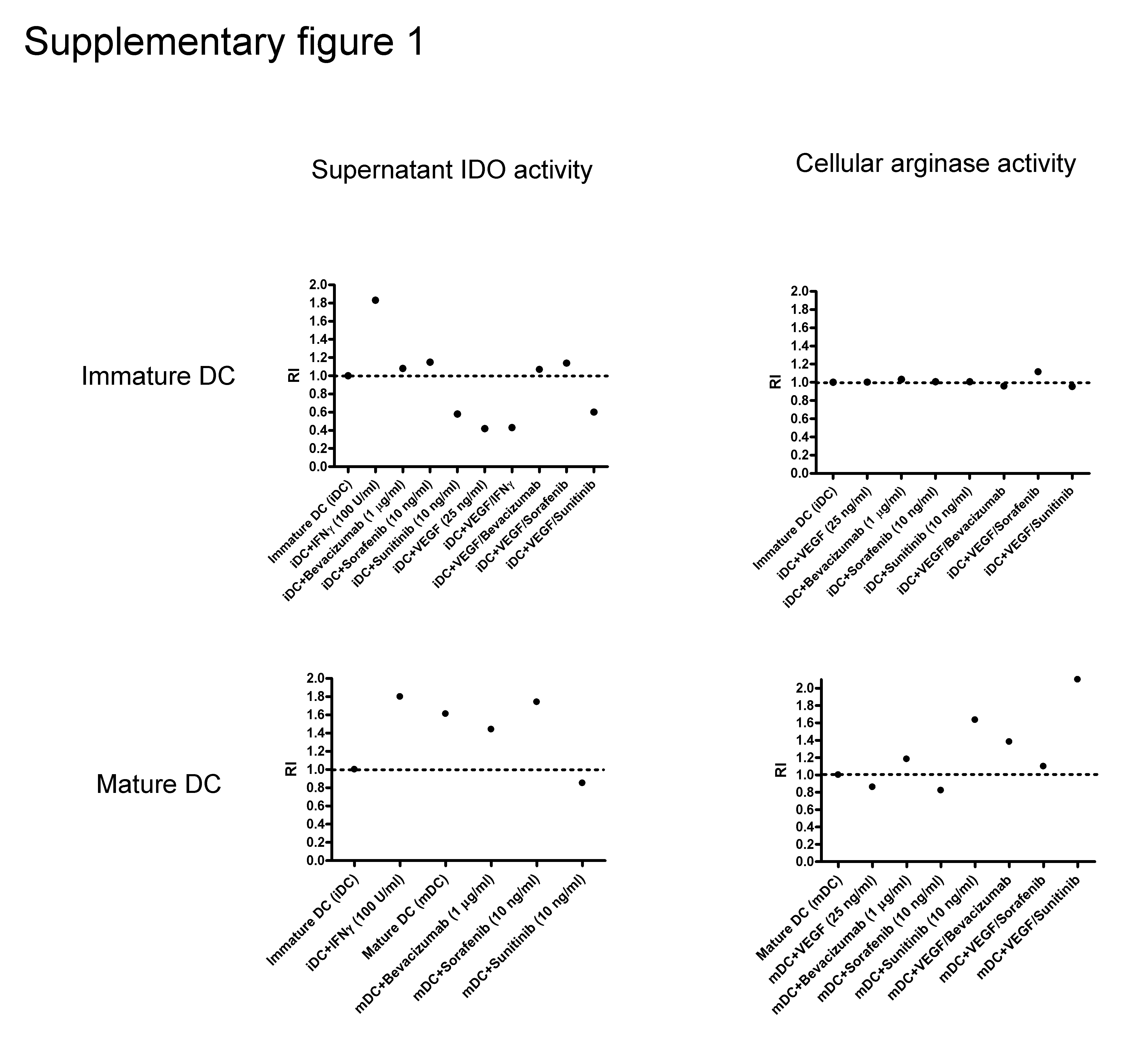

Supplement: Supplementary Figure 1 [file 6604965x1.tif]

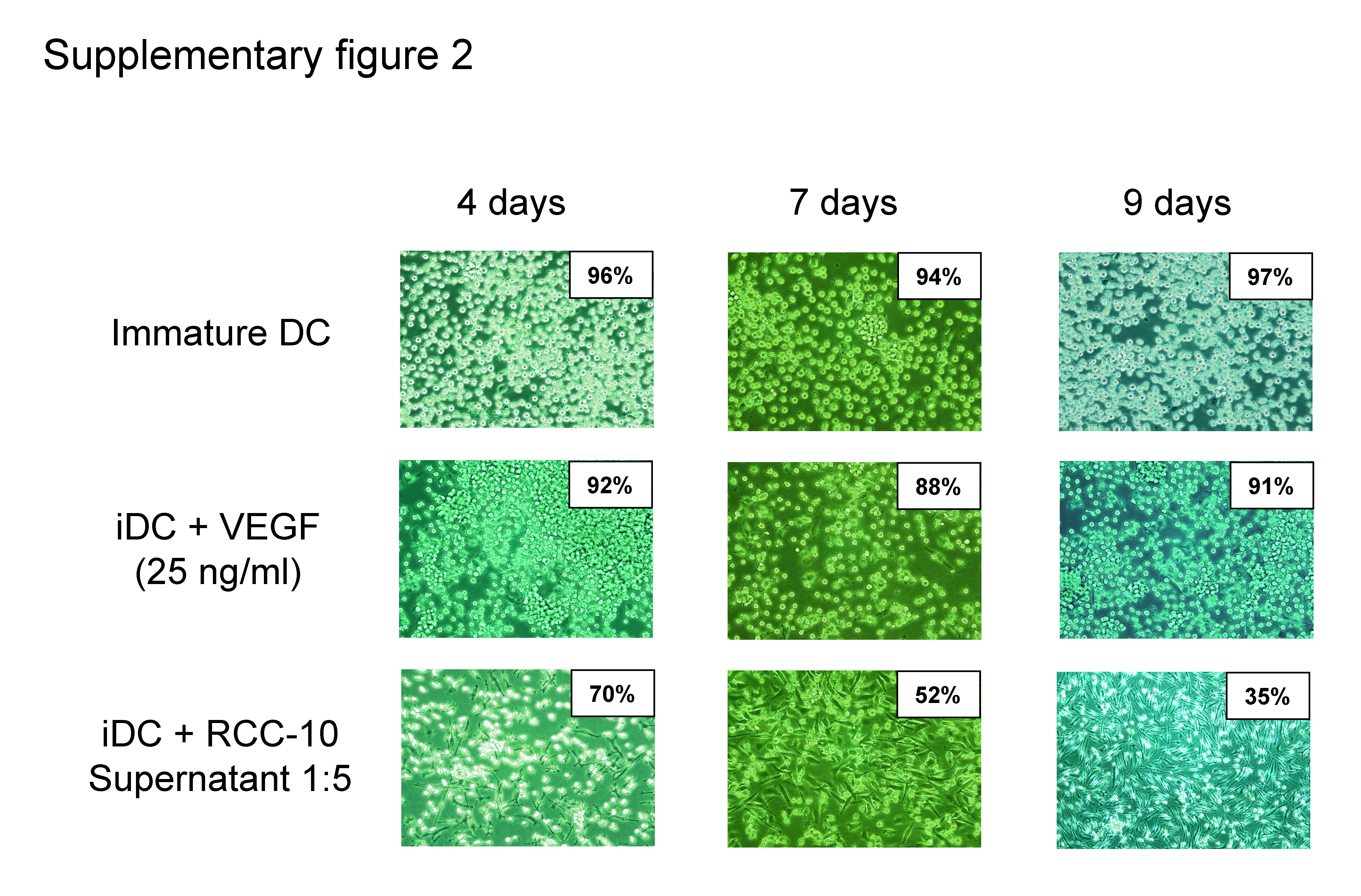

Supplement: Supplementary Figure 2 [file 6604965x2.tif]

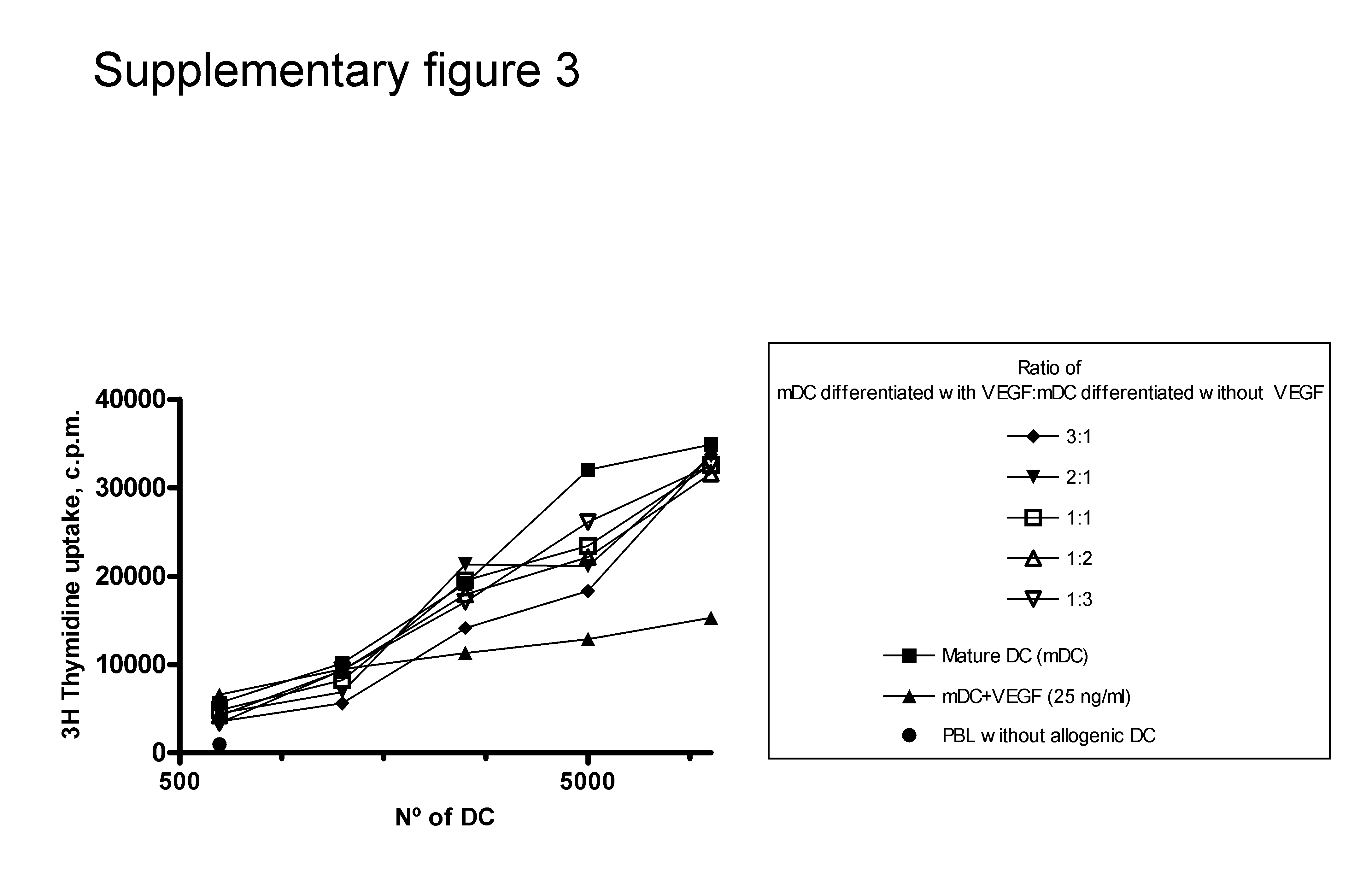

Supplement: Supplementary Figure 3 [file 6604965x3.tif]

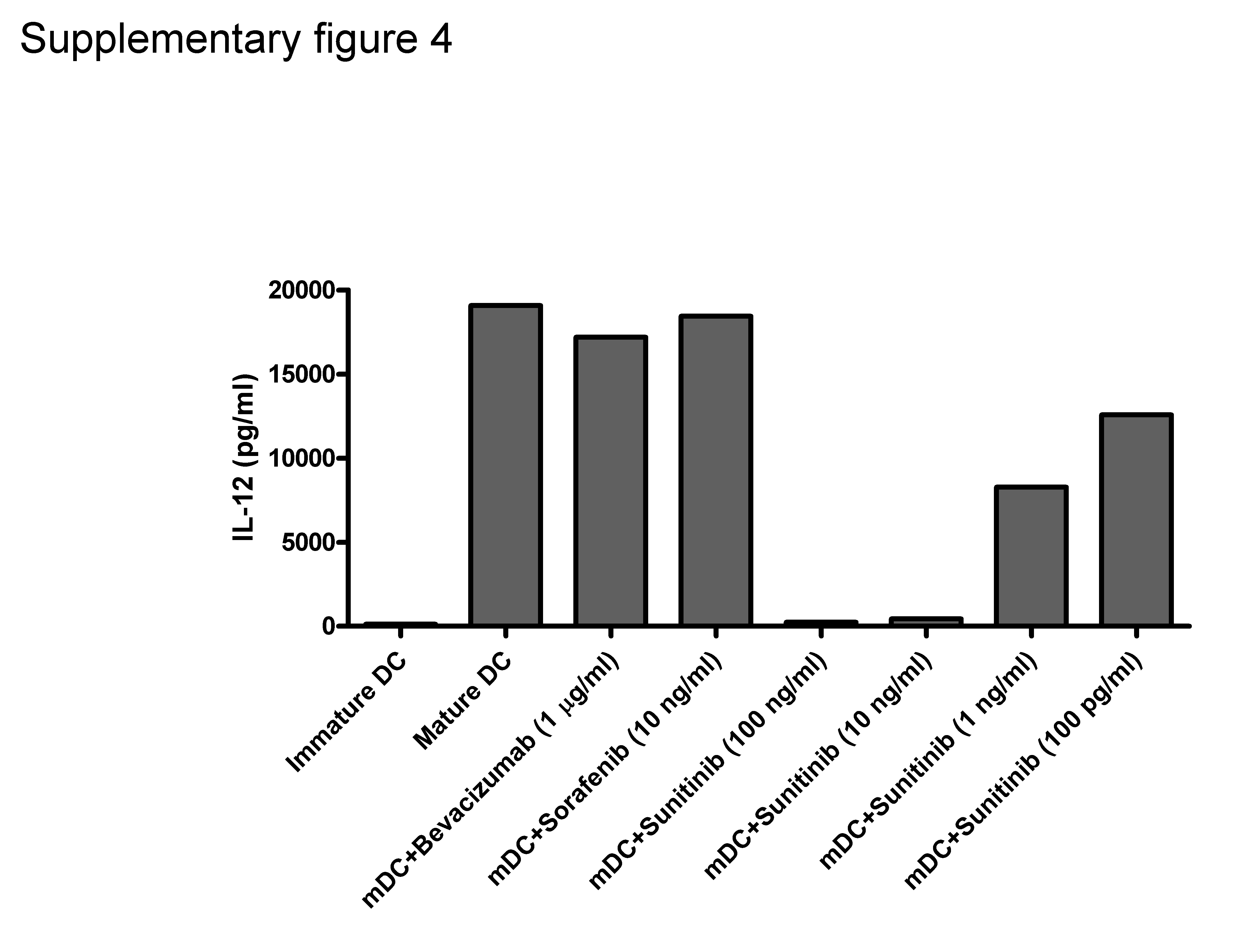

Supplement: Supplementary Figure 4 [file 6604965x4.tif]

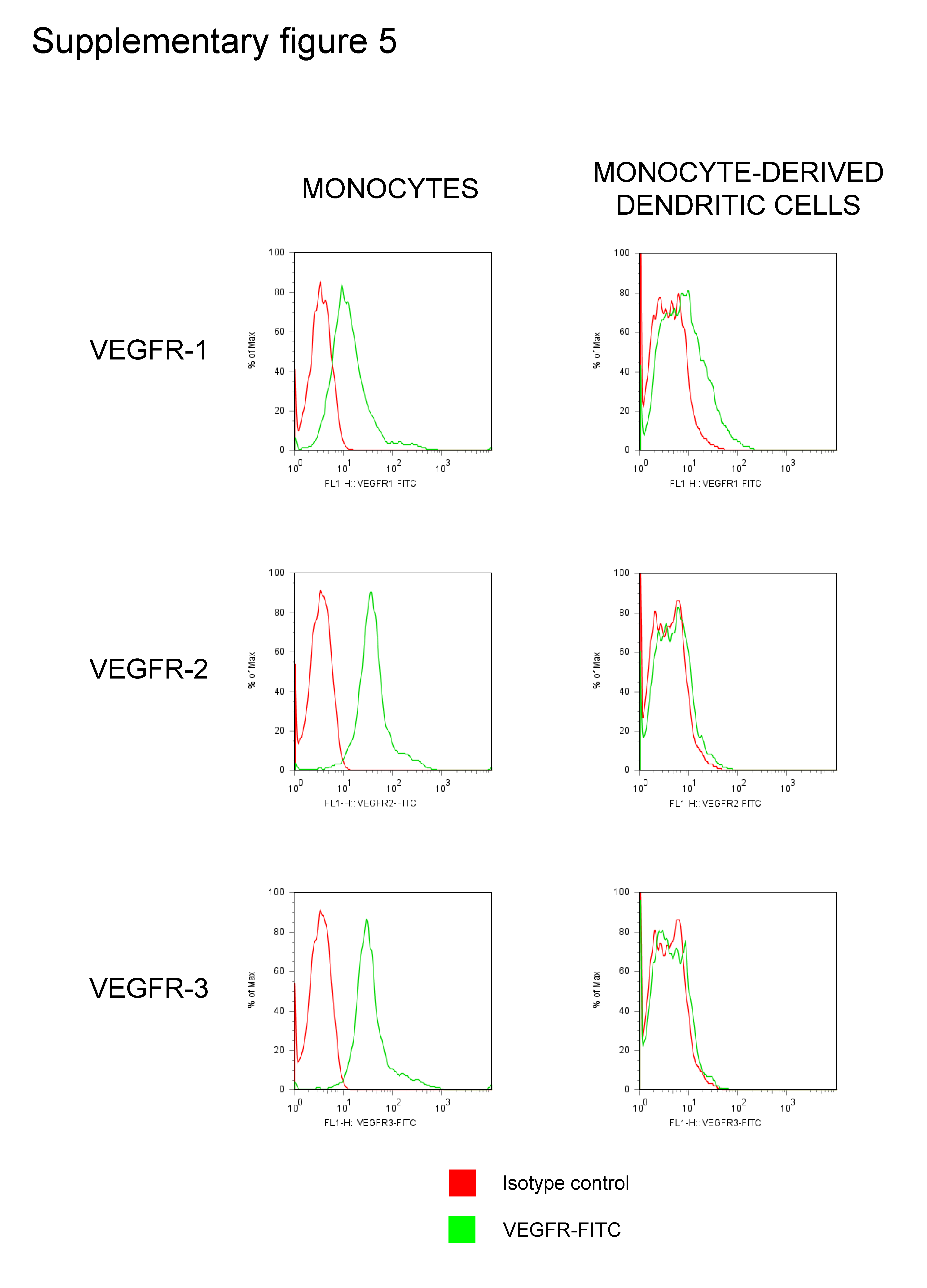

Supplement: Supplementary Figure 5 [file 6604965x5.tif]
