# Supplementary material for: Influence of bevacizumab, sunitinib and sorafenib as single agents or in combination on the inhibitory effects of VEGF on human dendritic cell differentiation from monocytes
Source: Br J Cancer. 2009 Mar 10;100(7):1111–9. doi: 10.1038/sj.bjc.6604965 (PMC2670006; doi:10.1038/sj.bjc.6604965)
Supplement: Supplementary Figures Legends [file 6604965x6.doc]

**Supplementary figure 1**

**VEGF added to DC differentiation cultures does not increase IDO or arginase.**

IDO was studied by kynurenine production normalized by [kynurenine]/[tryptophan] in the supernatant of DC differentiated under the influence of the indicated compounds. Arginase was measured in cell lysates by a colorimetric assay measuring urea production. Bevacizumab and sorafenib, added at the indicated concentrations, normalized IDO activity when VEGF had been added whereas sunitinib increased by itself arginase activity. For normalization, a relation index (RI) was calculated as [kynurenine]/[tryptophan] in dendritic cells in the experimental conditions divided by the same parameter in control dendritic cells without VEGF or RCC supernatants during differentiation. DC were matured with TNF-α, IFN-α and poly I:C or IFN-γ when indicated.

**Supplementary figure 2**

VEGF, RCC-10 supernatants during differentiation of monocytes to DC change adherence and morphology under phase contrast microscopy. As it can be seen RCC-10 but not VEGF changed the morphology towards a more spread and adherent phenotype. Percentages of floating versus adherent cells are included for each condition.

**Supplementary figure 3**

DC differentiated with VEGF did not repress MLR stimulating activity when added at different ratios to DC:T co-cultures.

**Supplementary figure 4**

Effects on IL-12 production of lower concentrations of sunitinib, than those shown in figure 5 under identical conditions to promote maturation with TNF-α, IFN-α and poly I:C.

**Supplementary figure 5**

FACS analysis by indirect immunofluorescencce with specific mAb of surface VEGFRs expression on monocytes and DC derived from monocytes
